# Neural correlate of human reciprocity in social interactions

**DOI:** 10.3389/fnins.2013.00239

**Published:** 2013-12-17

**Authors:** Shiro Sakaiya, Yuki Shiraito, Junko Kato, Hiroko Ide, Kensuke Okada, Kouji Takano, Kenji Kansaku

**Affiliations:** ^1^Center for Social Research and Data Archives, Institute of Social Science, The University of TokyoTokyo, Japan; ^2^Department of Politics, Princeton UniversityPrinceton, NJ, USA; ^3^Graduate School of Law and Politics, The University of TokyoTokyo, Japan; ^4^Graduate School of Humanities and Social Sciences, University of TsukubaTsukuba, Japan; ^5^Department of Psychology, Senshu UniversityKawasaki, Japan; ^6^Systems Neuroscience Section, Department of Rehabilitation for Brain Functions, Research Institute of National Rehabilitation Center for Persons with DisabilitiesTokorozawa, Japan

**Keywords:** cooperation, reciprocity, theory of mind, reward, default mode network, amygdala, DLPFC, VMPFC

## Abstract

Reciprocity plays a key role maintaining cooperation in society. However, little is known about the neural process that underpins human reciprocity during social interactions. Our neuroimaging study manipulated partner identity (computer, human) and strategy (random, tit-for-tat) in repeated prisoner's dilemma games and investigated the neural correlate of reciprocal interaction with humans. Reciprocal cooperation with humans but exploitation of computers by defection was associated with activation in the left amygdala. Amygdala activation was also positively and negatively correlated with a preference change for human partners following tit-for-tat and random strategies, respectively. The correlated activation represented the intensity of positive feeling toward reciprocal and negative feeling toward non-reciprocal partners, and so reflected reciprocity in social interaction. Reciprocity in social interaction, however, might plausibly be misinterpreted and so we also examined the neural coding of insight into the reciprocity of partners. Those with and without insight revealed differential brain activation across the reward-related circuitry (i.e., the right middle dorsolateral prefrontal cortex and dorsal caudate) and theory of mind (ToM) regions [i.e., ventromedial prefrontal cortex (VMPFC) and precuneus]. Among differential activations, activation in the precuneus, which accompanied deactivation of the VMPFC, was specific to those without insight into human partners who were engaged in a tit-for-tat strategy. This asymmetric (de)activation might involve specific contributions of ToM regions to the human search for reciprocity. Consequently, the intensity of emotion attached to human reciprocity was represented in the amygdala, whereas insight into the reciprocity of others was reflected in activation across the reward-related and ToM regions. This suggests the critical role of mentalizing, which was not equated with reward expectation during social interactions.

## Introduction

Cooperation is a critical component of human social behavior. Compared with other social animals, humans engage in cooperative behaviors in a wide variety of social contexts (Hammerstein, 2003; Stevens and Hauser, 2004). In the prisoner's dilemma (PD) game, which has been used to examine cooperative behavior theoretically, the Nash equilibrium refers to mutual defection, which results in a lower payoff for each player than does mutual cooperation and so poses a dilemma. The situation simulated by the PD game can be replicated in society. Rational consideration is expected to lead individual members of a group, society, and international community to defect and result in a shortage of public goods and damages collective well-being in the long run (Olson, 1965). In reality, however, human cooperation is ubiquitous. Reciprocity plays a key role maintaining cooperation in society in which individuals expect to encounter others repeatedly (Axelrod and Hamilton, 1981; Henrich et al., 2003). Indeed, recent behavioral studies reported that humans are conditional cooperators who discriminately cooperate with cooperators, but not with non-cooperators (Wedekind and Milinski, 2000; Fehr and Fischbacher, 2003; Fehr and Camerer, 2007). Reciprocity, which is unique to human society (Clutton-Brock, 2009; Connor, 2010; Melis and Semmann, 2010), is intimately connected with high social cognition (Brosnan et al., 2010) and is expected to relate to the workings of the human brain (Fehr and Rockenbach, 2004; Yamasue et al., 2009). However, the brain activity that underpins human reciprocity during social interaction has not been explored fully.

To explore the neural process behind human reciprocity, we investigated the brain activation during repeated PD games in which we manipulated the experimental conditions related to the game [i.e., partner identity (computer or human) and strategy (random or tit-for-tat)] in a 2 × 2 factorial design. Published studies that manipulated the identity of partners have found more activation of theory of mind (ToM; the ability to represent the mental states of self and others) regions when games were played against humans than against computers and reported different implications of the reward circuitry between playing with humans and computers (McCabe et al., 2001; Rilling et al., 2002, 2004; Krach et al., 2008). However, the differential activation might not necessarily have been attributable ultimately to partner identity: differential activation might also be linked with differences in observed and expected behaviors of partners with distinct identities. To differentiate their effects on brain activation, we therefore manipulated both the identity and strategy of partners. Recent experiments that focused on the effect of partner strategies explored differential activation in the dorsolateral prefrontal cortex (DLPFC) when playing with human agents who were cooperative, neutral, and non-cooperative (Suzuki et al., 2011) and found activation in the superior temporal sulcus as a function of successful adaption to reciprocal/non-reciprocal strategies of computer agents (Haruno and Kawato, 2009). In distinction, we focused on a neural process that would be associated specifically with reciprocity from humans (i.e., not with computers) and cross-examined the neural processes while controlling both identity and strategy.

Whereas neuroimaging studies using games originally focused on reward-related regions [the caudate nucleus, the orbitofrontal cortex (OFC), the anterior and posterior cingulate cortex, and the DLPFC] (Lee, 2008), an increasing number of studies (McCabe et al., 2001; Rilling et al., 2002, 2008a; Fehr and Rockenbach, 2004; Lee, 2006; Rilling and Sanfey, 2011) have reported activation of brain regions implicated in ToM, including the temporal parietal junction and the precuneus (Carrington and Bailey, 2009; Van Overwalle and Baetens, 2009). The ventromedial prefrontal cortex (VMPFC) and the amygdala have been often implicated in both ToM and reward processing (Lee, 2006; Beckmann et al., 2009; Roy et al., 2012). The decision to cooperate/defect (CD) requires one to make an inference from the behavior of others in a variety of contexts and to revise and change one's own behavior based on the anticipated reactions of others and is expected to accompany activations in ToM regions. Going beyond functional localization, experimental games have recently reported the representation of subjective values relating to inference of other's behaviors and insight into opponent's strategies in ToM region (Hampton et al., 2008; Bhatt et al., 2010; Carter et al., 2012). Whereas these published experiments used games that had no possibility of cooperation, we used repeated PD games that involved the possibility of cooperation and thus reciprocity. This enabled us to explore the neural representation of insight into reciprocity of partner strategy and subjective values attached to reciprocal and non-reciprocal partners.

The neural processes underlying reciprocity in social interaction should involve both emotion and social cognition (van den Bos et al., 2009; Strobel et al., 2011). We hypothesized that the presence and absence of reciprocity in social interaction with humans involved different emotional states, which are represented by subjective values, i.e., preferences for partners, in the amygdala. The presence and absence of reciprocity, however, may be plausibly misinterpreted, particularly when inference and insight into the intent of others is required for such an interpretation. We hypothesized that those with and without insight into reciprocity would reveal differential activation across reward-related and ToM regions and that the differential activation was not a mere reflection of reward expectation. Specifically, insight into human reciprocity has great consequences for our social life. Therefore, we also hypothesized that the neural activity specific to inferring reciprocity from humans would involve regions that have been linked to mentalizing, such as the VMPFC and precuneus.

## Materials and methods

### Subjects

In total, 26 healthy volunteers (14 females, 12 males; mean age ± SD, 20.5 ± 2.1) recruited from university campuses were screened to exclude those inappropriate for magnetic resonance (MR) scanning. All participants were neurologically normal and strongly right-handed according to the Edinburgh Inventory (Oldfield, 1971). Participants completed all four sessions and complied with the movement restrictions during functional magnetic resonance imaging (fMRI) sessions. Prior to scanning, participants finished a computer tutorial, and we confirmed that they fully understood the procedure. All subjects provided written informed consent for the study. This study complied with the Code of Ethics of the World Medical Association (Declaration of Helsinki) and was approved by the Ethics Committee of the National Rehabilitation Center for Persons with Disabilities.

### Procedure and design

One female and one male researcher conducted all experiments together to control for the putative influence of gender of the experimenter on the playing behavior of participants (Skotko et al., 1974).

**Figure 1:**
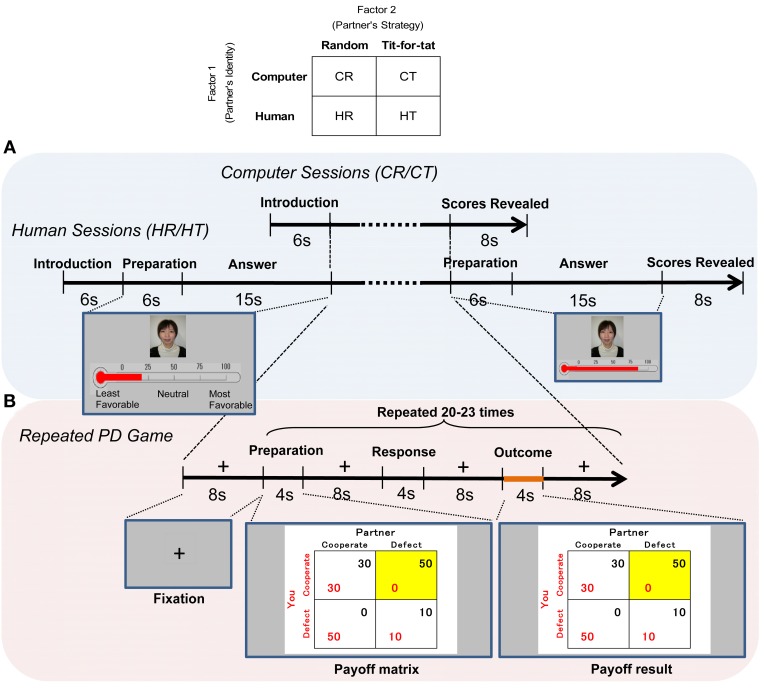
**Time course of the fMRI experiment. (A)** After their partner was introduced, the subjects played 20–23 rounds of the prisoner's dilemma game, and both players' scores for the session were displayed on a screen for 8 s at the end of each of four sessions (CR/CT/HR/HT). Before and after the games with humans, the subjects viewed a picture of a same-sex partner displayed on the screen for 6 s and rated their preference on a thermometric scale for her/him for 15 s. The same picture was shown in both human (HR/HT) sessions. **(B)** During each round, the subjects viewed a payoff matrix for 4 s, pressed a key for 2 s, and viewed the outcome for 4 s. The cell of the payoff matrix chosen in the round was highlighted. Imaging analysis was focused on the outcome stage. The stages were separated by an 8-s display of a fixation cross.

Experiments followed a factorial design with two factors and two levels: a partner's putative identity (computer vs. human) and a partner's controlled strategy (random vs. tit-for-tat). Four sessions were conducted, and data were collected under four contextual conditions (Figure 1): computer-random (CR), computer-tit-for-tat (CT), human-random (HR), and human-tit-for-tat (HT). Participants engaged in each of the four sessions in random order. Each session ended after 20–23 rounds (randomly assigned) of PD games, and participants were told that each session could terminate at any point before 30 rounds were played. Each session continued for approximately 15–20 min, with a break of 3–5 min between sessions. Partners' responses were generated by a computer algorithm. A random strategy was programmed to involve cooperation (defection) with a probability of 0.5 in each round. A tit-for-tat strategy was programmed to start with cooperation and then repeat the response given by the subject in the previous round (Axelrod and Hamilton, 1981).

Participants were told that their final reward would be proportional to the total sum of their payoffs from the games. Participants were informed about the partner's identity (i.e., computer or human), but not informed about the partner's strategy (i.e., the way in which a partner was programmed to play). Participants were photographed and explained that their pictures were shown to the same-sex human partner, whereas they were shown the partner's picture: the partner was allegedly playing in another room and was not introduced in person. Using a post-scanning questionnaire, we asked the subjects whether they had noticed any inconsistencies or differences between the actual playing of the game and the explanation prior to the experiments. All of them answered that they were playing, as had been explained, two sessions with humans and the computer, respectively; they believed that they were playing with actual humans in human sessions.

### fMRI paradigm

At the beginning of all sessions, the subjects were informed about the identity of their partner, and this introductory stage was the same in both the computer and human sessions (see Figure 1A for details). Before and after playing games in each of the human (HR/HT) sessions, the subjects viewed a picture of a same-sex human partner and scaled their preferences using a thermometric scale (explained in detail in *Behavioral Data*). The same picture was shown in both human sessions. During a round of PD games in each of four sessions (Figure 1B), subjects chose to cooperate (C) or defect (D). Four outcomes were shown in the form of a payoff matrix: cooperate/cooperate (CC), cooperate/defect (CD), defect/cooperate (DC), and defect/defect (DD), where the first letter represents the response of participants and the second represents the response of partners. The cell of the payoff matrix chosen in the round was highlighted.

### Behavioral data

We used two behavioral indicators for behavioral and neural analyses: changes in preference for human partners and insight into reciprocity of partner strategy. Changes in preference were aimed to quantify the participant's affection toward partners that was changed by playing games. Before and after each human (HR/HT) session, the subjects were asked to indicate their preferences. Preference was rated on a thermometric scale, on which 0° means “least favorable,” 100° means “most favorable,” and 50° means “neutral” (Figure 1A). The thermometric scale has been widely used to measure voter preferences for candidates in elections (Weisberg and Miller, 1979; Cairns et al., 2006), and was used in a published fMRI study (Kato et al., 2009). We subtracted the pre-session from the post-session rating and calculated changes in preferences. Changes in preferences were used to calculate correlations with other behavioral indicators, including the player's cooperation/defection rates and session scores (represented by the average payoff per round in each session). We also calculated their correlations with brain activation, as explained in more detail in *Neural Activity and Behavior*.

Insight into reciprocity of partner strategy was a behavioral indicator by which subjects in each session were split into two. We divided the participants into two groups according to their answer to a simple question that was focused on a critical difference between the two strategies; i.e., the presence and absence of reciprocity. After the fMRI sessions, participants who had been kept uninformed about partner strategy completed a separate questionnaire for each session to determine whether they felt that their partner had responded to their (the participants') choices. The insight group consisted of participants who answered “no” to this question for the random (CR/HR) sessions and “yes” to this question for the tit-for-tat (CT/HT) sessions. Those who answered in the opposite directions were assigned to the no-insight group. For behavioral analysis, this categorization was used as a dependent variable in logistic regression analysis that had partner's identity and strategy as independent variables. We also used it as a factor in a Three-Way analysis of variance (ANOVA) of imaging data, as explained in more details in *Imaging Analysis*.

Behavioral and psychometric analyses were conducted using the IBM SPSS Statistics (ver. 19) and Stata (ver. 11.1) software.

### Image acquisition

The experiment was conducted in an MR scanner (Exelart, Toshiba) at the research institute. The stimulus was presented and synchronized with the MR scanner using Presentation (Neurobehavioral Systems, San Francisco, CA, USA). During sessions, gradient echo T2^*^-weighted echo-planar images with BOLD contrast were acquired at 1.5 T (TR/TE = 3000/40 ms, *FA* = 85°, slice thickness/gap = 6/2 mm, *FOV* = 25 × 25 cm^2^, matrix size = 64 × 64, 18 slices). Each session consisted of 256 scans, the first three of which were discarded to allow for T1 equilibration effects. T1-weighted structural images were also acquired after the four sessions were completed.

### Imaging analysis

Imaging data were preprocessed and analyzed using SPM5 (Statistical Parametric Mapping 5; Wellcome Department of Imaging Neuroscience, Institute of Neurology, University College London, UK). Realignment processing assured that the participants moved their heads less than 2 mm. The realigned images were then normalized to a Montreal Neurological Institute EPI template and smoothed with an 8-mm full-width-at-half-maximum Gaussian kernel. A high-pass filter with a cut-off period of 128 s was applied to remove low-frequency noise, and an autoregressive (order one) model was used to correct for short-range serial correlations.

We obtained individual-level contrast images during the outcome stage (4 s; Figure 1B), in which subjects reviewed their payoff vis-à-vis partners', since we focused on the neural correlate of reciprocity. A general linear model (GLM) was used to estimate the parameters for each experimental condition (CC, CD, DC, or DD) in four sessions (CR/CT/HR/HT). The GLM also included six additional regressors of no interest to model head movement. These regressors allowed movement effects to be discounted when looking for brain activation.

We used contrasts for each individual for the second-level analysis and estimated activation at group level. Data from 104 sessions = 26 subjects × 4 contextual conditions (CR/CT/HR/HT) were analyzed by factorial (between-subjects) ANOVA (Jackson, 2011). We performed two kinds of Three-Way ANOVA. The first model included the three factors of partner's identity, strategy, and payoff outcome [identity (computer/human) × strategy (random/tit-for-tat) × outcome (CC/CD/DC/DD)]. In the first model, we aimed to examine outcome-related activation that would vary with the identity and strategy of the partner. The second ANOVA model combined the three factors of partner's identity, strategy, and insight into reciprocity in partner strategy [identity (computer/human) × strategy (random/tit-for-tat) × insight (insight/no-insight)]. The second model enabled us to differentiate activation between those with and without insight into strategy while controlling the identity and strategy of partner. We applied a threshold of *p* < 0.05, corrected for family-wise error (FWE), for multiple comparisons across the whole brain to an activation map with the threshold of an uncorrected *p* < 0.001, combined with a cluster-size threshold of 10 voxels. Because the statistical power for detecting an interacted activation is generally low (von Eye and Schuster, 1998), we identified the appropriate regions via a *post-hoc* confirmation of statistically significant activation using contrast estimates.

We calculated contrast estimates using MarsBaR (ver. 0.43) and analyzed inferences to examine their statistical significance using IBM SPSS Statistics (ver. 19) and Stata (ver. 11.1). We used rfxplot (revision 19) (Gläscher, 2009) to generate fitted responses.

Activated regions were anatomically labeled using WFU PickAtlas (ver. 3.03, Wake Forest University School of Medicine) and SPM Anatomy Toolbox (ver. 1.8, Institute of Neuroscience and Medicine) and located visually using an anatomical atlas (Naidich et al., 2009). All *x*, *y*, and *z* coordinates are reported in MNI space.

### Neural activity and behavior

We used preference changes as behavioral indicators to examine the correlation with brain activation using contrast estimates. We calculated the correlation between changes in self-rated preference for human partners during each session and the individual activation in the amygdala that was associated with the entire session; i.e., estimated without a separate regressor for each round outcome. Activation patterns that were dissociated between the insight and no-insight groups were also compared for each session.

## Results

### Behavioral analysis

#### Subjects cooperated more frequently in human and tit-for-tat sessions than otherwise

During the game sessions, participants chose to cooperate in an average of 58.5% of the rounds. Cooperation rates and proportions of payoff outcomes in each session were averaged over 26 participants and are summarized in Supplementary Table 1. A 2 × 2 (identity × strategy) ANOVA on cooperation rates revealed a main effect of partner identity [*F*_(1, 25)_ = 13.98, *p* < 0.01]. Cooperation with a human partner (64.4 ± 4.2%, mean ± SE of trials) was more frequent than that with a computer partner (52.7 ± 4.8%). We also found a main effect of strategy [*F*_(1, 25)_ = 50.47, *p* < 0.001]. Cooperation in tit-for-tat (CT/HT) sessions (74.1 ± 4.5%) was more likely than in random (CR/HR) sessions (43.0 ± 3.5%). The interaction between partner identity and strategy was not statistically significant.

#### Those with insight were found more in human and tit-for-tat sessions than otherwise

Table 1A shows the distributions of the insight and no-insight groups in each session. The logistic regression analysis (see Table 1B for details) showed that participants were more likely to have insight into reciprocity of partner strategy (*p* < 0.05) when playing with a human partner (i.e., HR/HT sessions) than when playing with a computer partner (i.e., CR/CT sessions). This analysis also revealed that participants tended to be more insightful (*p* < 0.01) in tit-for-tat (CT/HT) sessions than in random (CR/HR) sessions. The interaction between partner identity and strategy was not statistically significant.

#### Insight into reciprocity increased cooperation and payoff only in tit-for-tat sessions

Cooperation rates and proportions of payoff outcomes were significantly different between the insight and no-insight groups only in HT sessions. The cooperation rate in the no-insight group (48.5 ± 12.9%) was significantly lower [*t*_(24)_ = −3.32, *p* < 0.01] than that in the insight group (86.8 ± 5.1%). The rate of mutual cooperation (CC) in the no-insight group (34.3 ± 13.7%) was significantly lower [*t*_(24)_ = −3.04, *p* < 0.01] than that in the insight group (80.6 ± 7.3%), whereas the rate of mutual defection (DD) in the no-insight group (36.4 ± 13.1%) was significantly higher [*t*_(24)_ = 3.47, *p* < 0.01] than that in the insight group (6.0 ± 3.0%). In addition, session scores of the insight and no-insight groups revealed no significant differences across the four sessions [*t*_(102)_ = −1.53, *p* = 0.13] and involved no consistent difference when playing against a distinct strategy: the session scores in the no-insight group were higher [*t*_(24)_ = 2.11, *p* < 0.05] than those in the insight group in random sessions, but were significantly lower [*t*_(24)_ = −3.78, *p* < 0.001] than those in the insight group in tit-for-tat sessions. This result was consistent with the expectation that those with insight were more cooperative and rewarded more by playing games than those without insight only in tit-for-tat sessions. This also indicates that the behavioral indicator successfully distinguished those with insight from those without.

#### Preferences were correlated with cooperation in tit-for-tat but with payoff in random sessions

We also conducted correlation analyses on the relationship between changes in preference for partners and other behavioral indicators in human (HR/HT) sessions (see Table 1C for details). Preference change was significantly correlated with rate of cooperation/defection only in HT sessions: subject's cooperation rate (*r* = 0.429, *p* < 0.05) and partner's defection rate (*r* = −0.436, *p* < 0.05). We also examined the correlation between payoff outcome and preference change in each session. In HR sessions, preference change was negatively correlated with the rate of unilateral cooperation (CD; *r* = −0.394, *p* < 0.05). In HT sessions, preference change was positively correlated with the rate of mutual cooperation (CC; *r* = 0.496, *p* < 0.05) but negatively correlated with that of unilateral cooperation (CD; *r* = −0.532, *p* < 0.01) and unilateral defection (DC; *r* = −0.545, *p* < 0.01). Additionally, a preference change was significantly correlated with session scores in HR sessions only: subject's session score (*r* = 0.390, *p* < 0.05), partner's session score (*r* = −0.398, *p* < 0.05), and relative gain (subject's session score/partner's session score; *r* = 0.417, *p* < 0.05).

Table 1**Results of behavioral analyses**.

**Table:** **A. Distribution of subjects with and without insight**.

**Session**	**Insight**	**No-insight**	**Total**
Computer-Random	5	21	26
Computer-TFT	15	11	26
Human-Random	12	14	26
Human-TFT	20	6	26

**Table:** **B. Logistic regression (Dependent Variable: Insight = 1, No-insight = 0)**.

**Variable**	**Coef**.	***SE***	***Z***
Identity (Computer = 0, Human = 1)	1.59	0.63	2.51^*^
Strategy (Random = 0, Tit-for-tat = 1)	1.75	0.64	2.74^**^
Identity × Strategy	−0.70	0.88	−0.79
Constant	−1.44	0.50	−2.88^**^

N = 104, Pseudo R^2^ = 0.133.

** p < 0.01;

* p < 0.05.

**Table:** **C. Correlations with changes in preferences**.

	**HR session**	**HT session**
Subject's Cooperation Rate	−0.361	0.429^*^
Partner's Defection Rate	−0.018	−0.436^*^
Mutual Cooperation (CC) Rate	−0.251	0.496^*^
Unilateral Cooperation (CD) Rate	−0.394^*^	−0.532^**^
Unilateral Defection (DC) Rate	0.374	−0.545^**^
Mutual Defection (DD) Rate	0.216	−0.263
Subject's Session Score	0.390^*^	0.360
Partner's Session Score	−0.398^*^	0.366
Relative Gain	0.417^*^	−0.244

Subjects with insight into their partner's strategy were those who regarded that their partners had not responded to their choices for the random (CR/HR) sessions and that their partners had responded to their choices for the tit-for-tat (CT/HT) sessions.

** p < 0.01,

* p < 0.05.

### Imaging analysis

#### Mutual cooperation with humans but unilateral defection with the computer was associated with activation in the amygdala

**Figure 2:**
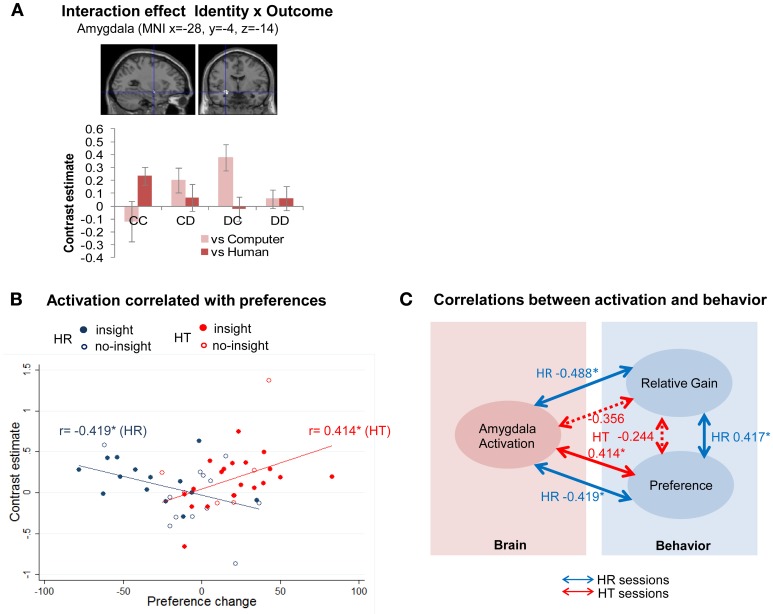
**Amygdala activation was positively and negatively correlated with preference changes for human partners following tit-for-tat and random strategies, respectively. (A)** Amygdala activation was linked to mutual cooperation with humans and to unilateral defection from a computer [(−28, −4, −14), cluster-level FWE corrected *p* < 0.05]. Bar plots represent contrast estimates ± standard errors of the peak coordinates for each of the outcomes. **(B)** Participants with higher activation represented by contrast estimates at (−28, −4, −14) [cluster-level FWE corrected *p* < 0.05] showed increased preference for a partner following a tit-for-tat strategy (*r* = 0.414, *p* = 0.036), but decreased preference for one following a random strategy (*r* = −0.419, *p* = 0.033); ^*^p < 0.05. Each plot represents an individual participant. Those without insight are indicated by hollow circles. The solid blue and red lines represented the fitted regression line of all cases, i.e., those with and without insight in HR and HT, respectively. **(C)** Individual activation in the amygdala was negatively correlated with an individual's relative gain (subject's session score/partner's session score) against a human partner following a random strategy (HR) (*r* = −0.488, *p* = 0.012), whereas no statistically significant correlation was observed when playing with a human following a tit-for-tat strategy (HT) (*r* = −0.356, *p* = 0.075). Note that a partial correlation between activation of the amygdala and relative gain was not statistically significant (*r* = −0.272, *p* = 0.189) in the HR session when the effect of preference change was controlled. Dotted lines represent correlations that were not statistically significant. ^*^p < 0.05.

We found that outcome-related activation varied with the identity of the partner in Three-Way ANOVA [identity (computer/human) × strategy (random/tit-for-tat) × outcome (CC/CD/DC/DD)], as represented in interaction effect (identity *×* outcome) (Supplementary Table 2). An interaction effect was detected for a specific contrast (human^*CC* > *DC*^ > computer^*CC* > *DC*^) in the amygdala [(−28, −4, −14) cluster-level FWE corrected *p* < 0.05; *k* = 291] (Figure 2A). The amygdala was associated with mutual cooperation with humans but unilateral defection with the computer, which implied different psychological processes linked to cooperation/defection with computers/humans. A *post-hoc* examination of contrast estimates also found an interaction effect (strategy *×* outcome), below a threshold of multiple comparisons, in the right anterior DLPFC (Supplementary Table 2; Supplementary Figure 1).

#### Positive and negative subjective values were represented in the amygdala during reciprocal and non-reciprocal interactions

Individuals' different activations in the left amygdala (−28, −4, −14) in human (HR/HT) sessions were examined against subjective values, i.e., individual self-rated changes in preferences for partners. The activation was significantly negatively correlated with changes in preference in HR sessions (*r* = −0.419, *p* < 0.05) and significantly positively correlated with these changes in HT sessions (*r* = 0.414, *p* < 0.05) (Figure 2B).

We further examined the activation against other behavioral indicators to determine whether the relationship between the neural activity and preference changes was valid. Amygdala activation was significantly negatively correlated with relative gain (subject's session score/partner's session score) in HR sessions (*r* = −0.488, *p* < 0.05), but not HT sessions (*r* = −0.356, *p* = 0.075) (Figure 2C). The partial correlation between amygdala activation and relative gain was not statistically significant (*r* = −0.272, *p* = 0.189) in HR sessions when the effect of preference change was controlled. Thus, the simple correlation between amygdala activation and relative gain in HR sessions may be spurious. The neural correlate of preference changes was not significantly different when participants did and did not gain insight into the reciprocity of partners (Figure 2B). In HR sessions, correlations were negative in both the insight and no-insight groups (*N* = 14, *r* = −0.349, *p* = 0.222 and *N* = 12, *r* = −0.350, *p* = 0.265, respectively). In HT sessions, correlations were positive both in the insight and no-insight groups (*N* = 20, *r* = 0.438, *p* = 0.053 and *N* = 6, *r* = 0.524, *p* = 0.286). These correlations were statistically non-significant (*p* > 0.05), probably due to the limited data, but indicated that the absence of insight did not necessarily interfere with the relationship between amygdala activation and changes in preferences.

#### Those with and without insight into reciprocity revealed differential activation across reward circuitry and ToM regions

A Three-Way ANOVA [identity (computer/human) × strategy (random/tit-for-tat) × insight (insight/no-insight)] found differential activation between those with and without insight into strategy, while controlling the identity and strategy of partner (see Table 2 for detailed results). Precuneus activation between the insight and no-insight groups was varied with strategy, which was represented in a significant interaction (strategy × insight) effect [BA7; (−14, −72, 32), FWE-corrected *p* < 0.01]. Insight into partner's strategy (insight > no-insight) was associated with significantly greater activity in the dorsal caudate [(6, 10, 16), FWE-corrected *p* < 0.01], VMPFC [BA32/10; (−10, 32, −4), FWE-corrected *p* < 0.05], and right middle DLPFC [BA46/9; (44, 18, 26), FWE-corrected *p* < 0.05]. Significant activation observed in the dorsal caudate and VMPFC was specific to the insight group. In contrast, significant activation in the left DLPFC [BA46; (−44, 30, 10), FWE corrected *p* < 0.05] was specific to the no-insight group. In addition to these regions in the reward circuitry and/or ToM regions, ANOVA revealed activation in the occipital cortex (Supplementary Table 3). These results demonstrate that both reward-related and ToM regions were linked to gaining insight into the partner's reciprocity during games.

**Table 2:** **Results of Three-Way Factorial ANOVA (Identity × Strategy × Insight)**.

Region	Brodmann's area	Mask	MNI Coordinates			*k*	*F*_(1, 311)_	*t*_(311)_
			*x*	*y*	*z*			
**MAIN EFFECT OF INSIGHT**								
Dorsal Caudate			6	10	16^**^	571	32.04	
VMPFC	BA32/10		−10	32	−4	229	23.03	
Right mid−DLPFC	BA46/9		44	18	26	571	22.99	
Left DLPFC	BA46		−44	30	10	96	22.02	
**INTERACTION EFFECT OF STRATEGY × INSIGHT**								
Precuneus	BA7		−14	−72	32^**^	776	28.01	
**INSIGHT > NO−INSIGHT**								
Dorsal Caudate		Exclusive	6	10	16^**^	576^^++^^		5.66
VMPFC	BA32/10	Exclusive	−10	32	−4^*^	318^^+^^		4.80
Right mid−DLPFC	BA46/9	Inclusive	44	18	26^*^	198		4.80
**NO−INSIGHT > INSIGHT**								
Left DLPFC	BA46	Exclusive	−44	30	10^*^	126		4.69

** FWE corrected p < 0.01,

* FWE corrected p < 0.05,

++ cluster-level corrected p < 0.01,

+ cluster-level corrected p < 0.05.

#### Representation of insight in the DLPFC was not affected by reward expectation

**Figure 3:**
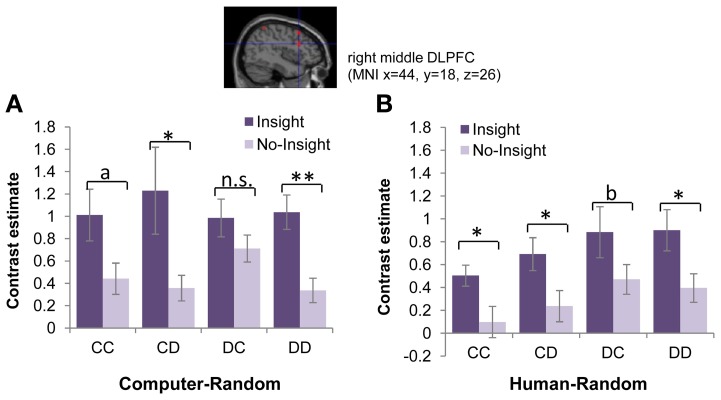
**Representation of insight into reciprocity of partner strategy in the right middle DLPFC**. Significant activation in the right middle DLPFC [(44, 18, 26), FWE corrected *p* < 0.05] distinguished the insight and no-insight groups when the partner's identity (computer or human) and payoff outcome were controlled. Bar plots represent mean comparisons of activation in the right middle DLPFC between the insight and no-insight groups for each payoff outcome. Error bars represent standard errors. ^**^p < 0.01; ^*^p < 0.05; ^a^*p* = 0.066; ^b^*p* = 0.067; n.s. = not significant (two-tailed *t*-test). **(A)** Computer-random (CR) session. **(B)** Human-random (HR) session.

Activation linked to insight might simply reflect the implications of reward processing, rather than represent insight into reciprocity in the DLPFC, which has been linked to general reasoning (Wood and Grafman, 2003; Van Overwalle, 2011), but also is a part of the reward circuitry. To explore this possibility, we examined whether differential activation was a function of payoff outcomes, which involved different material gains for subjects. Across different payoff outcomes (CC/CD/DC/DD), activation in the DLPFC was higher in the insight group than in the no-insight group for random (CR and HR) sessions (Figure 3). Two-sample *t*-tests of the contrast estimates for the right middle DLPFC region along outcomes showed consistently different activation patterns between the two groups in random sessions. No significant difference (*p* > 0.05) in activation, regardless of payoff outcomes, was found during tit-for-tat sessions or in the other regions implicated in random sessions.

#### The presence and absence of insight into human reciprocity was dissociated by precuneus activation and VMPFC deactivation

**Figure 4:**
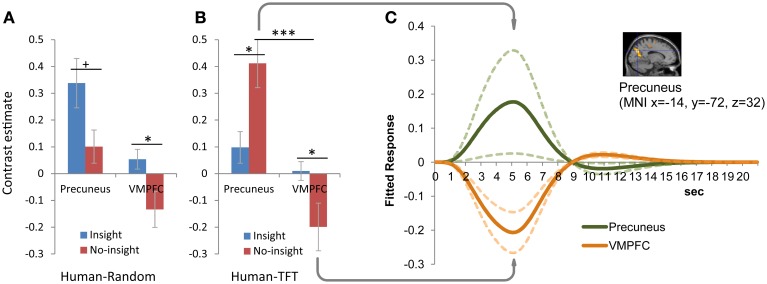
**Differential activation in the precuneus and VMPFC between those with and without insight into reciprocity of human partners. (A)** Human-random (HR) session. **(B)** Human-tit-for-tat (HT) session. Insight into reciprocity interacted with partner strategy in activation in the precuneus [(−14, −72, 32), FWE corrected *p* < 0.01], but was associated specifically with activation in the VMPFC [(−10, 32, −4), FWE-corrected *p* < 0.05]. The reverse association of activation with the presence and absence of insight [(Insight/No-Insight) × (precuneus/VMPFC); *F*_(1, 51)_ = 13.23, *p* < 0.001] was observed for HT, but not HR sessions. Bar plots represent mean comparisons of activation in the precuneus and VMPFC between the insight and no-insight groups. Error bars represent standard errors. ^***^p < 0.001; ^*^p < 0.05; ^+^*p* = 0.051. **(C)** Asymmetric pattern of activation in the precuneus and deactivation in the VMPFC was specific to the no-insight group during HT sessions. Time-series plots (solid lines) indicate the fitted response at the coordinates. Dotted lines represent standard errors.

Activation in the precuneus was associated with those who did not consider that partners were responding to their prior cooperation/defection (insight group in random sessions and no-insight group in tit-for-tat sessions). Figures 4A,B present the interaction (strategy × insight) of precuneus activation and VMPFC activation specific to the insight group, respectively, during the human random and tit-for-tat (HR and HT) sessions. In the HT session (Figure 4B) specifically, significant differential activation (*p* < 0.05) in two regions was reversely associated with the presence and absence of insight. Examination of contrast estimates of differential activation revealed a significant interaction [(Insight/No-Insight) × (precuneus/VMPFC); *F*_(1, 51)_ = 13.23, *p* < 0.001]. The cross-over pattern of activation along the presence and absence of insight satisfies a statistical criteria for “reversed association,” i.e., a “qualitative rather than quantitative difference in brain activity” to “dissociate” regions (Henson, 2005, 2006), which has been reported in recent experiments on social cognition (Raposo et al., 2011; Zaki et al., 2012; Izuma and Adolphs, 2013). Furthermore, dissociated activation in the precuneus in the no-insight group was accompanied by significant deactivation of the VMPFC, which involved an asymmetric pattern in fitted responses of (de)activation (Figure 4C).

For comparison, the neural activation pattern in the computer sessions was examined (Supplementary Figure 2). Activation in the precuneus along with insight was significantly different during HT sessions, but not in CT sessions, (Supplementary Figure 2B) and so did not satisfy the criteria for “reversed association.” Consequently, significant (de)activation that was dissociated across two regions was specific to those without insight into the reciprocity of human partners engaging in a tit-for-tat strategy.

## Discussion

### Reciprocity in social interaction was reflected in correlated activation in the amygdala

Activation in the left amygdala was associated with mutual cooperation (CC) with humans but unilateral defection (DC) against computers (Figure 2A, Supplementary Table 2). Positive feelings during reciprocal cooperation with humans, but negative feelings during exploitation of computers, may involve intense affective experiences, activating the amygdala in our experiment: published experiments reported amygdala activation when trust of and reciprocity with others was undermined (Rilling et al., 2008b; Rilling and Sanfey, 2011). The left amygdala, similar to the activated region in our study, is associated with more intense social stimuli (Kramer et al., 2007) and greater emotional intensity (Phan et al., 2004), whereas the amygdala has generally been linked with both moral (Moll et al., 2005) and social (Olsson and Ochsner, 2008) cognition.

To further explore the possibility of representation of intense subjective values in the amygdala, we examined individual differential activations and found the opposite tendency in activations across sessions. Amygdala activation was positively and negatively correlated with a preference change for human partners following tit-for-tat and random strategies, respectively (Figure 2B). This correlation was found across the insight and no-insight groups and was thus not affected by insight into reciprocity. Examination against behavioral data also demonstrates that the correlated activation did not result from different material gains during reciprocal and non-reciprocal interactions (Figure 2C). Combined, these results indicate that positive feelings during reciprocal interaction but negative feelings during non-reciprocal interaction may be involved in a stronger (i.e., more intense) emotional experience than *vice versa*, which was represented in the amygdala. Behavioral analysis further confirmed this interpretation. During reciprocal interaction with human partners using a tit-for-tat strategy, mutual cooperation contributed to increasing preference vis-à-vis both unilateral cooperation and unilateral defection associated with decreased preference. Changes in preferences toward non-reciprocal human partners with a random strategy were influenced only by unilateral cooperation among behavioral outcomes, which contributed to decreasing preferences.

The amygdala has been linked to either positive or negative emotion in some studies on social decisions (Adolphs, 2002; van't Wout and Sanfey, 2008; Haruno and Frith, 2010), but has also been linked to both positive and negative social stimuli, including rewards, in others (Costafreda et al., 2008). In contrast to published works, the opposite tendency in activation in our results conveys general implications for the role of the amygdala in representing the intensity of affective values. In studies of non-social affective values such as odor (Zald and Pardo, 1997; Anderson et al., 2003) and taste (O'Doherty et al., 2001; Small et al., 2003), the intensity of values was represented in the amygdala and dissociated from valence that has been linked to the OFC. However, representation of the intensity of affective values in the amygdala has not yet been examined directly in studies on social decision, in which experimenters may not be able to apply appropriate control over the correlations and confounding effects of intensity and valence (i.e., higher intensity for negative than for positive stimuli; higher intensity and greater valence for negative stimuli) (Anderson et al., 2003). Opposite associations of valence and intensity across sessions; i.e., higher intensity for negative feelings in non-reciprocal interaction but positive feelings in reciprocal interaction, enabled our experiment to distinguish two dimensions of affective values. The representation of intensity in the amygdala, distinct from valence, in a study of social affective values was reported for the first time. The result also demonstrated that correlated activation in the amygdala reflected the presence and absence of reciprocity in social interaction.

### Insight into reciprocity of partner strategy was represented in reward circuitry and ToM regions

Differential activation was found between those with and without insight into reciprocity in partner strategy across other reward-related and ToM regions than the amygdala. The right and left DLPFC were associated with the presence and absence of insight, respectively, while activation specific to insight was found in both the VMPFC and dorsal caudate (Table 2). A recent study reported that compliance with social norms during games activates regions in the VMPFC and the right middle DLPFC, both of which were associated with insight into reciprocity in our study (Baumgartner et al., 2011). The caudate nucleus has been implicated in trial-and-error reinforcement learning (Delgado et al., 2005), and the dorsal caudate has been associated with learning via positive and negative reinforcement and reward-seeking (O'Doherty et al., 2004; Tricomi et al., 2004; Delgado et al., 2005; Schonberg et al., 2007; Spitzer et al., 2007; Tricomi and Fiez, 2008). A region in the dorsal caudate that activated with the presence of insight in our study was implicated in a decision with uncertainty about fairness of partners in iterated trust games (Rao et al., 2013) and also with altruistic punishment of norm violation that involved no overt benefits (Strobel et al., 2011). These results have supported the possibility that activation across the reward and ToM regions might be interpreted as reflecting the involvement of subjective judgment of others rather than the expectation of reward directly from playing games.

### Representation of insight in the DLPFC was not interfered with by reward expectation

Those with insight gained significantly more from playing games than those without insight in tit-for-tat sessions, but not in random sessions (see *Behavioral Analysis*); consequently, differential activations along insight might not have resulted from a simple response to material gains. To examine whether activation is a mere reflection of reward-processing, we focused on the DLPFC, which is part of the reward circuitry, but is also linked to social cognition. A recent study (De Vico Fallani et al., 2010) using the EEG hyper-scanning technique (Dumas et al., 2011) has also reported activation in this region during reciprocal interaction in the iterated PD games. In our experiment, those with insight were expected to internalize their partner's behavioral patterns, whereas those without insight more likely continued to interpret information in an effort to understand how their partners were responding. Previous reports on the right and left DLPFC are consistent with this contrasting interpretation. More specifically, the right middle DLPFC, similar to the activated region among those with insight, has been linked to judgments about interpersonal relationships (Zink et al., 2008), as well as to compliance with norms (Spitzer et al., 2007), whereas the left DLPFC, again similar to the region associated with those without insight, has been linked to integrating information (Bunge et al., 2009) and to strategic sophistication (Yoshida et al., 2010) and deception (Baumgartner et al., 2009) during games.

We further examined activation in the right DLPFC as a function of payoff outcomes and found that insight representation was not affected by reward expectation. Insight into reciprocity was represented, irrespective of payoff outcomes, in activation in random sessions (Figures 3A,B). As expected, we did not observe significant differential activation in tit-for-tat sessions in which the effect of insight was confounded with the one of reward. Combined, our results imply that the neural representation of insight is independent of reward expectation.

### Contribution of ToM to searching for human reciprocity

Gaining insight into human reciprocity is critical for our social life. We found the brain activity that was specific to the human search for reciprocity in the VMPFC and precuneus. Although the VMPFC was thought to be generally associated with social cognition and mentalizing (Bush et al., 2000; Wood and Grafman, 2003), a region in the VMPFC that was activated among participants with insight in our study has been linked to inferences related to the intentions of others when playing games (Cooper et al., 2010). The precuneus was activated when subjects did not find partners to be responsive to their prior choices (either correctly or incorrectly, varied with the partner's actual strategies); i.e., among the insight group in random sessions and the no-insight group in tit-for-tat sessions (Table 2). Those without insight into reciprocity plausibly tried to discern the partner's behavioral patterns, which was consistent with the association of the precuneus with high-level integration of social cognition in general (Cavanna and Trimble, 2006). Figures 4A,B contrast the significant activation pattern in the precuneus and VMPFC when human partners used random and tit-for-tat strategies, respectively. The cross-over activation along insight between the two regions in a human tit-for-tat session (Figure 4B) involved a reversed association that represented a “qualitative” difference in the brain activity and dissociated the regions along conditions (i.e., the presence and absence of insight) (Henson, 2005, 2006). Previous experiments have found activation in both the VMPFC and precuneus during games in which participants thought that they were playing with humans rather than with computers (McCabe et al., 2001; Rilling et al., 2004; Krach et al., 2008). Distinct from this, our results indicated their further specialization in ToM. Indeed, the precuneus and VMPFC have been functionally dissociated in recent literature on the default-mode network (DMN) (Uddin et al., 2009), which is presumably activated during rest (Raichle et al., 2001) and overlaps with the ToM region (Schilbach et al., 2008; Spreng et al., 2009). The precuneus has been linked to attempts to understand the responsiveness of others, whereas the VMPFC has been linked to reasoning on true or false beliefs about reality (Sommer et al., 2007; van Buuren et al., 2010).

Those without insight into human reciprocity revealed dissociated activation, which involved asymmetric (de)activation in the precuneus and the VMPFC (Figure 4C). Whereas those without insight into a tit-for-tat strategy were less cooperative and rewarded than those with insight across sessions (see *Behavioral Analysis*), the significant (de)activation was observed among only those without insight into the reciprocal response of humans, but not of computers (Supplementary Figure 2). These results specifically implicate the asymmetric (de)activation in searching for human reciprocity. A recent study of a ToM-related task (i.e., episodic memory retrieval) (Sestieri et al., 2011) dissociated (de)activation of the precuneus and VMPFC, which were similar to activated regions in this study (Spreng and Grady, 2010). Taken together, the asymmetric (de)activation of the precuneus and VMPFC among those without insight into human reciprocity suggests a contribution of ToM regions specific to inferring and searching for human reciprocity.

In summary, the intensity of emotion during reciprocal and non-reciprocal interaction with humans was represented in the amygdala, whereas insight into the reciprocity of others was reflected in activation across the reward-related and ToM regions. These results indicate the critical role of mentalizing, which was not equated with reward expectation during social interactions that involved the possibility of cooperation.

### Author contributions

Shiro Sakaiya, Yuki Shiraito, Junko Kato, Hiroko Ide, and Kenji Kansaku designed research; Shiro Sakaiya, Yuki Shiraito, and Kouji Takano performed research; Shiro Sakaiya, Yuki Shiraito, Junko Kato, and Kensuke Okada analyzed data; Junko Kato wrote the paper.

### Conflict of interest statement

The authors declare that the research was conducted in the absence of any commercial or financial relationships that could be construed as a potential conflict of interest.
