# Supplementary material for: Neural correlate of human reciprocity in social interactions
Source: Front Neurosci. 2013 Dec 17;7:239. doi: 10.3389/fnins.2013.00239 (PMC3865425; doi:10.3389/fnins.2013.00239)
Supplement: Supplementary file 1 [file DataSheet1.DOCX]

**Supplementary Figure 1. Interaction of payoff outcomes with strategy (random/tit-for-tat) in the right anterior DLPFC**

Activation was linked to defection when playing against a random strategy and cooperation when playing against a tit-for-tat strategy in the anterior part of the right DLPFC ([30, 42, 40], uncorrected *p* < 0.001; *k* = 15).

**Supplementary Figure 2. Differential activation in the precuneus and VMPFC between those with and without insight into reciprocity of computer partners**

**A** Computer-random (CR) session. **B** Computer-tit-for-tat (CT) session. Error bars represent standard errors. ** *p* < 0.01; * *p* < 0.05

**Supplementary Table 1. Behavioral Outcomes in Each Session (Average for 26 Subjects)**

|  | Session | | | |
| --- | --- | --- | --- | --- |
|  | Computer-Random | Computer-TFT | Human-Random | Human-TFT |
| % CX | 35.1 | 70.2 | 50.9 | 78.0 |
| % CC | 14.9 | 62.5 | 27.1 | 69.9 |
| % CD | 20.2 | 7.7 | 23.7 | 8.1 |
| % DC | 32.7 | 9.3 | 25.8 | 9.0 |
| % DD | 32.2 | 20.4 | 23.4 | 13.0 |

*Note*:

Four outcomes (CC, CD, DC, and DD) mean cooperate/cooperate, cooperate/defect, defect/cooperate, and defect/defect, respectively, where the first letter represents the response of participants and the second represents the response of partners.

% CX represents % CC plus % CD, or subjects’ cooperation rate.

**Supplementary Table 2. Results of Three−way Factorial ANOVA (Identity×Strategy×Payoff Outcome)**

|  |  | |  | **MNI Coordinates** | | | |  |  |  |  |  |  |
| --- | --- | --- | --- | --- | --- | --- | --- | --- | --- | --- | --- | --- | --- |
| **Region** | | | **Brodmann Area** | **x** | **y** | **z** |  | **k** |  | **F_(1,311)_** | **t_(311)_** | **uncorrected p** |  |
|  | |  |  |  |  |  |  |  |  |  |  |  |  |
| **Interaction effect of Identity x Outcome** | | | | |  |  |  |  |  |  |  |  |  |
|  | Amygdala | |  | −28 | −4 | −14 | ^a^ | 35 |  | 6.26 |  | <0.001 |  |
|  |  | |  |  |  |  |  |  |  |  |  |  |  |
| **Human^CC>DC^ > Computer^CC>DC^** | | | | |  |  |  |  |  |  |  |  |  |
|  | Amygdala | |  | −28 | −4 | −14 |  | 291 | ^*^ |  | 4.00 | <0.001 |  |
|  |  | |  |  |  |  |  |  |  |  |  |  |  |
| **Interaction effect of Strategy x Outcome** | | | | |  |  |  |  |  |  |  |  |  |
|  | right anterior−DLPFC | | BA9/8 | 30 | 42 | 40 | ^b^ | 12 |  | 5.99 |  | <0.001 |  |
|  |  | |  |  |  |  |  |  |  |  |  |  |  |
| **Random^CX>DX^ > TFT^CX>DX^** | | | |  |  |  |  |  |  |  |  |  |  |
|  | right anterior−DLPFC | | BA9/8 | 30 | 42 | 40 | ^c^ | 15 |  |  | 3.29 | <0.001 |  |
| ^*^ cluster-level corrected p<0.05 | | |  |  |  |  |  |  |  |  |  |  |  |

*Note*:

CX and DX mean that subjects cooperate and defect, respectively, regardless of their partner’s cooperation/defection.

a: In examination of contrast estimates at the peak voxel and for the left amygdala, interaction effect was statistically significant (respectively, F(3, 315) = 4.927, p < 0.01 and F(3, 315) = 3.762, p < 0.05).

b: In examination of contrast estimates at the peak voxel and for the activated cluster, interaction effect was statistically significant (respectively, F(3, 315) = 6.116, p < 0.001 and F(3, 315) = 6.159, p < 0.01).

c: In examination of contrast estimates at the peak voxel and for the activated cluster, interaction effect was statistically significant (respectively, F(1,319) = 9.703, p < 0.01, F(1, 319) = 9.215, p < 0.005). Simple effects at the peak voxel of outcomes of CC/DD and CD/DD in both the random and TFT sessions were also statistically significant (p < 0.05).

**Supplementary Table 3. Results of Three-way Factorial ANOVA (Identity×Strategy×Insight) in the Occipital Cortex**

|  |  |  |  | **MNI Coordinates** | | |  |  |  |  |  |  |
| --- | --- | --- | --- | --- | --- | --- | --- | --- | --- | --- | --- | --- |
| **Region** | | **Brodmann Area** | **Mask** | **x** | **y** | **z** |  | **k** |  | **F_(1,311)_** | **t_(311)_** | **uncorrected p** |
|  |  |  |  |  |  |  |  |  |  |  |  |  |
| **Main effect of Insight** | |  |  |  |  |  |  |  |  |  |  |  |
|  | Occipital cortex | BA18 |  | 8 | −102 | 8 | ^**^ | 455 |  | 54.52 |  | <0.001 |
|  | Occipital cortex | BA19 |  | −26 | −72 | −6 | ^**^ | 1386 |  | 31.05 |  | <0.001 |
|  |  |  |  |  |  |  |  |  |  |  |  |  |
| **Insight > No−Insight** | |  |  |  |  |  |  |  |  |  |  |  |
|  | Occipital cortex | BA18 | exclusive | 8 | −102 | 8 | ^**^ | 287 | ^++^ |  | 7.83 | <0.001 |
|  | Occipital cortex | BA19 | inclusive | −26 | −72 | −6 | ^**^ | 1756 | ^++^ |  | 5.57 | <0.001 |

^**^ FWE corrected p<0.01 ^++^cluster-level corrected p<0.01

*Note*: The occipital cortex has been reported to be activated in ToM studies [Carrington & Bailey, 2009] and a recent study found that the core networks extend further into the occipital cortex than previously thought [Spreng et al., 2009].
